# A Phase II Study of Berzosertib in Combination with Carboplatin Compared with Docetaxel with Carboplatin in Metastatic Castration-Resistant Prostate Cancer

**DOI:** 10.1158/2767-9764.CRC-26-0488

**Published:** 2026-09-18

**Authors:** Atish D. Choudhury, Caiwei Zhong, Wanling Xie, Bose S. Kochupurakkal, Peter I-Fan Wu, Irbaz Bin Riaz, Ruolin Liu, David D. Yang, Edmund Folefac, Daniel Lee, Mamta Parikh, David J. Einstein, Elizabeth R. Kessler, Tina Mayer, Rana R. McKay, Amanda Pace, Kent W. Mouw, Li Chen, Viktor A. Adalsteinsson, Eliezer M. Van Allen, Charles A. Kunos, Steven D. Gore, Biswajit Das, Alan D’Andrea, Mary-Ellen Taplin, Geoffrey I. Shapiro

**Affiliations:** 1 Dana-Farber Cancer Institute, Boston, Massachusetts.; 2 Harvard Medical School, Boston, Massachusetts.; 3 Molecular Characterization Laboratory, Frederick National Laboratory for Cancer Research, Frederick, Maryland.; 4 Mayo Clinic, Phoenix, Arizona.; 5 Broad Institute of MIT and Harvard, Cambridge, Massachusetts.; 6 The Ohio State University Comprehensive Cancer Center, Columbus, Ohio.; 7 University of Pittsburgh, Pittsburgh, Pennsylvania.; 8 UC Davis Comprehensive Cancer Center, Sacramento, California.; 9 Beth Israel Deaconess Medical Center, Boston, Massachusetts.; 10 University of Colorado Hospital Cancer Center, Aurora, Colorado.; 11 Rutgers Cancer Institute of New Jersey, New Brunswick, New Jersey.; 12 University of California San Diego Moores Cancer Center, La Jolla, California.; 13 Markey Cancer Center, University of Kentucky, Lexington, Kentucky.; 14 Cancer Therapy Evaluation Program, National Cancer Institute, Rockville, Maryland.

## Abstract

**Purpose:**

Inhibitors of the ataxia-telangiectasia and Rad3-related (ATR) protein kinase, a critical component of the DNA damage repair response, have synergistic anticancer activity with platinum compounds in preclinical models. We therefore conducted a phase II study of the ATR inhibitor berzosertib + carboplatin versus docetaxel + carboplatin in metastatic castration-resistant prostate cancer (mCRPC).

**Patients and Methods:**

Patients previously treated with at least one androgen receptor pathway inhibitor and taxane underwent mandatory biopsy and were randomized 1:1 to receive arm A (docetaxel 60 mg/m^2^ + carboplatin AUC4 day 1 or carboplatin AUC5 if not a docetaxel candidate) or arm B (berzosertib 90 mg/m^2^ days 2 and 9 + carboplatin AUC5 day 1) every 21 days. The primary endpoint was overall response rate (ORR; ≥50% PSA decline or radiographic response).

**Results:**

Of 73 randomized patients, 65 received protocol treatment: 34 on arm A (26 docetaxel + carboplatin and 8 carboplatin alone) and 31 on arm B. Thirteen patients (38%) in arm A and 20 (65%) in arm B had ≥ grade 3 treatment-related adverse events (TrAE). ORR was 15% in arm A [5/34; 5/26 (19%) with docetaxel + carboplatin] and 0% in arm B (0/31). At interim analysis, after 65 of the planned 130 patients were treated, enrollment was halted due to futility. Homologous recombination repair (HRR) deficiency status from tissue and blood did not correlate with clinical outcomes, but ATM deficiency or high levels of phospho-Krüppel-associated box (KRAB) domain–associated protein 1 (pKAP1) detected in tissue were associated with clinical benefit across both arms of the trial.

**Conclusions:**

Berzosertib + carboplatin demonstrated lower ORR and more frequent ≥ grade 3 TrAEs compared with docetaxel + carboplatin in this heavily pretreated, biomarker-unselected population.

**Significance:**

The combination of the ATR inhibitor berzosertib with carboplatin demonstrated a lower ORR compared with docetaxel with carboplatin in a heavily pretreated biomarker-unselected mCRPC population. Assays for HRR deficiency showed limited predictive capability, but a combined tissue biomarker of ATM deficiency and elevated pKAP1 (indicating elevated replication stress) was associated with benefit in both study arms and warrants investigation for benefit from carboplatin-based therapy.

## Introduction

The genetic landscapes of primary prostate cancer (1) and metastatic castration-resistant prostate cancer (mCRPC; refs. 2, 3) have nominated oncogenic drivers and potential therapeutic targets. Despite extensive genetic characterization, clinical benefit from molecularly targeted agents other than those targeting the androgen receptor has so far been limited primarily to genetically defined subsets of patients with defects in DNA damage repair (DDR) pathways. The immune checkpoint inhibitors (ICI) pembrolizumab and dostarlimab are approved for use in microsatellite instability–high (MSI-H) or mismatch repair deficient (dMMR) solid tumors (4), and inhibitors of poly (ADP-ribose) polymerase (PARPi) have demonstrated clinical activity in biomarker-selected populations of patients with metastatic prostate cancer harboring deleterious alterations in genes involved in homologous recombination repair (HRR; refs. 5–8). However, only ∼2% to 3% of patients with mCRPC are phenotypically MSI-H/dMMR (9), and only ∼12% to 13% have mutations in *BRCA1* or *BRCA2* (10, 11)—even in these subpopulations, responses to ICIs (9, 12) and PARPis (13, 14) are not universal. As such, alternative paradigms for molecularly targeted therapy in mCRPC warrant exploration.

Understanding that DDR defects can represent a vulnerability in prostate cancer cells, we hypothesized that novel approaches to target these defects could provide clinical benefit to a larger proportion of patients. One such approach is through inhibiting the enzymatic activity of the ataxia-telangiectasia and Rad3–related (ATR) serine/threonine kinase. ATR is activated by replication protein A–coated single-stranded DNA, a nucleoprotein structure commonly generated at sites of DNA damage and stressed replication forks (15). Although the related kinase ATM is primarily involved in the response to DNA double-stranded breaks, ATR responds to a wide range of DNA damage and DNA replication problems. Thus, ATR inhibition is selectively toxic in cells with loss of genes involved in the DDR response (e.g., ATM) and nucleotide excision repair (e.g., XRCC1), as well as in cells reliant on alternative lengthening of telomeres or with high levels of replication stress induced by oncogenes (e.g., RAS isoforms, MYC, cyclin E) or stressors such as hypoxia.

Replication stress can also be induced exogenously through treatment with chemotherapeutics (16), and in preclinical studies, the ATR inhibitor berzosertib was found to be synergistic with a variety of DNA-damaging agents, most notably with cisplatin (17). The platinum compound carboplatin has demonstrated therapeutic benefit in patients with prostate cancer with known inherited *BRCA2* mutations (18, 19), as well as in “anaplastic” or “aggressive variant” prostate cancer (20), and is thus a rational combination partner for berzosertib. Importantly, platinum compounds can have activity in PARPi-resistant cancers (21–23). Additionally, it has been demonstrated that BRCA1-deficient cells rendered resistant to PARPi can be sensitive to ATR inhibition (24). These findings suggested that the combination of carboplatin with berzosertib would provide clinical benefit to patients with mCRPC with tumor homologous recombination deficiency (HRD), including those who received a prior PARPi, as well as to patients without tumor HRD due to the potential synergistic activity of berzosertib with carboplatin related to the induction of replication stress. We thus designed a randomized phase II trial to compare the response rate of this combination against a standard docetaxel + carboplatin combination regimen (25) in a biomarker-unselected population of patients with mCRPC.

## Patients and Methods

### Study design and patient selection

This was a randomized open-label phase II study of berzosertib + carboplatin versus docetaxel + carboplatin in patients with mCRPC. Eligible patients were adults (age ≥18 years) with Eastern Cooperative Oncology Group (ECOG) performance status (PS) 0 to 2, previously treated with at least one androgen receptor pathway inhibitor and taxane, with adequate renal, bone marrow, and liver function. Patients had castrate levels of testosterone (<50 ng/dL) and were evaluable for response either by baseline PSA ≥ 2 ng/mL or measurable disease by RECIST 1.1 criteria. Patients were required to have metastatic disease by conventional imaging and be willing to undergo a core biopsy of a recurrent/metastatic lesion adequate for next-generation sequencing (NGS) prior to trial entry (although confirmation of the adequacy of this biopsy material for NGS was not required for trial registration). There was no limit on the prior number of therapies. Prior treatment with a PARPi was permitted although patients previously treated with carboplatin or an ATR inhibitor were excluded.

The protocol was approved by the NCI Central Institutional Review Board, followed by local activation at Experimental Therapeutics Clinical Trials Network (ETCTN) sites (NCI/Cancer Therapy Evaluation Program protocol #10191, Dana-Farber/Harvard Cancer Center protocol # 19-715, NCT03517969). This study was conducted in accordance with the Declaration of Helsinki, International Council for Harmonisation Good Clinical Practice Guidelines, and other applicable guidelines, laws, and regulations. All participating patients signed informed consent before enrollment.

### Study endpoints

The primary endpoint of the study was the overall response rate (ORR) defined as either a PSA reduction greater than 50% or a confirmed radiographic response by RECIST 1.1. Key secondary endpoints included radiographic progression-free survival (rPFS), progression-free survival (PFS) by Prostate Cancer Working Group 3 (PCWG3) criteria (defined as when the treating physician felt the patient was “no longer clinically benefiting” from therapy—generally understood as radiographic or clinical/symptomatic progression; ref. 26), time to PSA progression by PCWG2 criteria (27), safety and adverse events, and the relationship of HRD detected from baseline tumor biopsy with clinical endpoints. Exploratory endpoints included overall survival (OS), ORR after cross-over from arm A to arm B (i.e., from the docetaxel-containing arm to the berzosertib-containing arm), and the relationship of additional exploratory tissue- and blood-based biomarkers with clinical endpoints.

The primary comparison of response rate and other efficacy endpoints was performed in an intent-to-treat analysis. For time-to-event endpoints, participants alive without achieving the event were censored at the date of the last evaluation. Treatment-related adverse event (TrAE) was defined as any AE with attribution of “possible,” “probable,” or “definite” relationship to study treatment or any grade 5 AE that occurred within 30 days of the last administration of drugs.

### Study procedures and assessments

Enrolled patients underwent mandatory pretreatment biopsy and were randomized 1:1 to receive arm A (docetaxel 60 mg/m^2^ + carboplatin AUC4 day 1; ref. 25) or arm B (berzosertib 90 mg/m^2^ days 2, 9 + carboplatin AUC5 day 1) every 21 days (28). Patients randomized to arm A who were not candidates for docetaxel (due to allergy or intolerance, grade 2 neuropathy, or ECOG PS = 2) received carboplatin AUC5 on day 1 as monotherapy. Stratification factors were (i) prior PARPi (yes vs. no) and (ii) evaluable disease by RECIST 1.1 (yes vs. no), with a *post hoc* analysis by whether or not the tumor harbored a pathogenic “HRR” mutation (as defined in “Whole-exome sequencing” below) detected in the mandatory pretreatment tumor biopsy. Restaging radiologic measurements, as determined through cross-sectional imaging of the chest, abdomen, and pelvis, as well as bone scintigraphy, were performed prior to every third cycle [i.e., prior to cycle 4 day 1 (C4D1), C7D1]. Patients on arm A were permitted to cross over to arm B (berzosertib + carboplatin) at the time of PSA progression [by PCWG2 criteria (27)] or radiographic progression (by PCWG3 criteria) if they remained clinically stable and continued to meet the eligibility criteria of the protocol. For patients on arm A who crossed over, imaging studies were obtained within 30 days of C1D1 (cross-over) and then prior to C4D1 (cross-over), C7D1 (cross-over), and so on. Patients were treated on study until progression by PCWG3 criteria, unequivocal clinical progression, unacceptable side effects, withdrawal of consent, or death.

### Statistical analyses

For the primary endpoint, we assumed an ORR of 20% in the docetaxel + carboplatin arm (29) and 40% in the berzosertib + carboplatin arm among patients with two or more prior therapies. A sample size of 65 patients per arm (*N* = 130) would provide 80% power to distinguish an ORR of 40% versus 20% using a one-sided *χ*^2^ test (α = 0.05).

The study was monitored for futility with one interim analysis, planned at approximately 50% accrual (i.e., 65 response-evaluable patients). If the observed ORR was lower in the berzosertib + carboplatin arm among this population, the study would be stopped early for lack of efficacy. The probability of early stopping was 0.50 under the null hypothesis and 0.04 under the alternative hypothesis. East software version 6 (Cytel Inc.) was used for sample size and interim monitoring considerations.

Time-to-event curves for rPFS, PFS, time to PSA progression, and OS were generated using the Kaplan–Meier method, with treatment effects [hazard ratios (HR) and 95% confidence intervals (CI)] estimated from Cox regression models. Comparison of PFS by tissue and plasma biomarker groups was conducted using the two-sided log-rank test.

All analyses were performed using SAS Software version 9.4 (SAS Institute Inc., RRID: SCR_008567).

### Nucleic acid extraction from tissue

Formalin-fixed tissue from mandatory preprotocol therapy biopsies was used for nucleic acid extraction. Tumor tissue was processed and embedded upon receipt at the NCI Early-Phase and Experimental Clinical Trials Biospecimen Bank. Slides were cut from 1 to 2 cores, and the first section was stained with hematoxylin and eosin for pathology quality control review to assess tumor content; unstained slides were macrodissected when needed, and then DNA and RNA were coextracted, aliquoted, and stored in a −80°C freezer until distribution for testing.

### Whole-exome sequencing

Tumor–germline whole-exome sequencing (WES) was performed through the Molecular Characterization (MoCha) laboratory at the Frederick National Laboratory for Cancer Research. Sequencing reads were processed with the MoCha in-house bioinformatics pipeline (https://github.com/FNL-MoCha/nextgenseq_pipeline). Raw base call files were demultiplexed and converted to FASTQ format with bcl2fastq (Illumina, version 2.18; RRID:SCR_015058), and read quality was assessed using FASTQC (RRID:SCR_014583). For WES, reads were aligned to the hg19 build of the human reference genome with the Burrows–Wheeler Aligner (BWA; RRID:SCR_010910), and the resulting BAM files were processed according to the Genome Analysis Toolkit (GATK; RRID:SCR_001876) best-practice workflow. Somatic variants were subsequently identified using GATK MuTect2 (RRID:SCR_026692). (One patient for whom germline WES failed was analyzed using a tumor-only pipeline to call somatic mutations by filtering out known single-nucleotide polymorphisms and variants present at >1% frequency in the gnomAD database https://gnomad.broadinstitute.org/.) We defined “HRD” based on the presence of a deleterious or likely deleterious mutation in a gene within the union of the “HRR” gene lists in the TALAPRO-2 (7) and PROfound (13) studies (excluding *PPP2R2A*): *BRCA1*, *BRCA2*, *ATM*, *BARD1*, *BRIP1*, *CDK12*, *CHEK1*, *CHEK2*, *FANCA*, *FANCL*, *NBN*, *PALB2*, *RAD51*, *RAD51B*, *RAD51C*, *RAD51D*, and *RAD54L*, understanding that many of these DDR genes are not involved in the core HRR pathway. Deleterious variants were defined as two-copy loss, truncation, frameshift, or single-nucleotide polymorphism that was categorized as pathogenic in ClinVar (https://www.ncbi.nlm.nih.gov/clinvar/; RRID:SCR_006169), deleterious by SIFT (https://sift.bii.a-star.edu.sg/; RRID:SCR_012813), or probably damaging by PolyPhen-2 (http://genetics.bwh.harvard.edu/pph2/; RRID:SCR_013189).

### Immunohistochemistry

One pair of serial sections of formalin-fixed, paraffin-embedded (FFPE) tumor biopsy samples was used for the RAD51 assay, and another pair was used for the ATM-Replication Stress (ATM-RS) assay. The pairs of slides were stained using antibodies to RAD51 [custom formulated (30)], GMNN (Leica; Geminin-L-CE; RRID:AB_563738) for the RAD51 assay, ATM (Abcam; ab32420, RRID:AB_725574), and phospho-Krüppel-associated box (KRAB) domain–associated protein 1 (pKAP1; Abcam; ab133440, RRID:AB_3712532) for the ATM-KAP1 assay. Staining for GMNN, ATM, and pKAP1 was performed on the Leica BOND, and RAD51 staining was performed on the Ventana DISCOVERY automated staining platforms using proprietary reagents. To determine HRD status of the sample, the RAD51 assay slides were examined at 40× magnification under an Olympus BX41 microscope. Tumor cell nuclei with greater than five RAD51 foci were designated as RAD51 foci-positive, and a minimum of three 40× tumor fields containing RAD51 foci-positive cells was required to classify a sample as HRR-proficient. If this criterion was not met and ≥3% of the tumor cells were positive for GMNN staining, then the sample was classified as HRR-deficient; if <3% of cells were positive for GMNN, HRD status was deemed indeterminate. FFPE sections of irradiated and unirradiated HRR-deficient and proficient breast cancer cell lines were used as technical controls for the staining (31).

ATM-stained slides were microscopically examined at 40× magnification, and the samples were classified as ATM-normal (>95% positive), ATM-negative (<5% positive), and ATM-heterogeneous (>5% and <95%). ATM staining in stromal cells within the sample served as an *in situ* control (32). Slides stained with pKAP1 were scanned and analyzed using the Aperio digital pathology platform (Leica Biosystems). The Nuclear V9 algorithm with precalibrated cutoffs for staining intensity levels was used to estimate the percentage of positive cells. An FFPE section of human tonsil was used as the technical control for staining. The ATM-RS biomarker classified the samples into three groups: (i) low ATM activity, (ii) normal ATM activity, and (iii) high replication stress based on combining results from ATM staining and the percentage positivity of pKAP1. ATM-heterogeneous samples with a percentage pKAP1 positivity lower than that of ATM-negative samples were deemed to lack functional ATM activity. Irrespective of ATM status, samples with greater than the 75th percentile of percentage pKAP1 positivity among ATM-positive and ATM-heterogeneous cases were defined as those with high replication stress.

### Circulating cell-free DNA sequencing

Blood specimens collected in Streck cell-free DNA (cfDNA) tubes were processed to plasma and frozen per ETCTN standard operating procedures. Plasma was aliquoted and stored in a −80°C freezer until distribution for testing. Genomic DNA extracted from blood collected at the pretreatment time point and frozen plasma from C1D1, C1D1 (cross-over; for patients randomized to arm A), and off-study time points was shipped in batches by the ECTCN Biorepository to the Genomics Platform at the Broad Institute. Ultralow-pass whole-genome sequencing (ULP-WGS) and matched tumor/germline targeted panel sequencing using a prostate cancer–specific bait set were performed as previously described (33). Tumor fraction (TFx) was estimated using ichorCNA (RRID:SCR_024768; ref. 34). For patients with matched germline DNA available, the same library from ULP-WGS was selected in hybrid capture using a custom targeted panel with a goal of 10,000× to 25,000× mean target coverage (MTC) depending on TFx, with 10,000× MTC for specimens with TFx >10% and 25,000× for <10%.

In addition, WGS was performed on matched pairs of cfDNA (median coverage, 38.7×; range, 19× to 75.3×) and buffy coat (median coverage, 12.4×; range, 9× to 29.8×) DNA samples. Raw base call files were demultiplexed and converted to FASTQ format with bcl2fastq (Illumina, version 2.18; RRID:SCR_015058). Reads were then aligned to the hg19 build of the human reference genome with BWA (RRID:SCR_010910). DirectHRD was applied following the published protocol (35), with higher HRD scores indicating a greater likelihood of true phenotypic HRD. The analyses were performed using the default HRD score cutoff of 1 as well as a more stringent cutoff of 2.5.

## Results

### Patients

**Figure 1.**
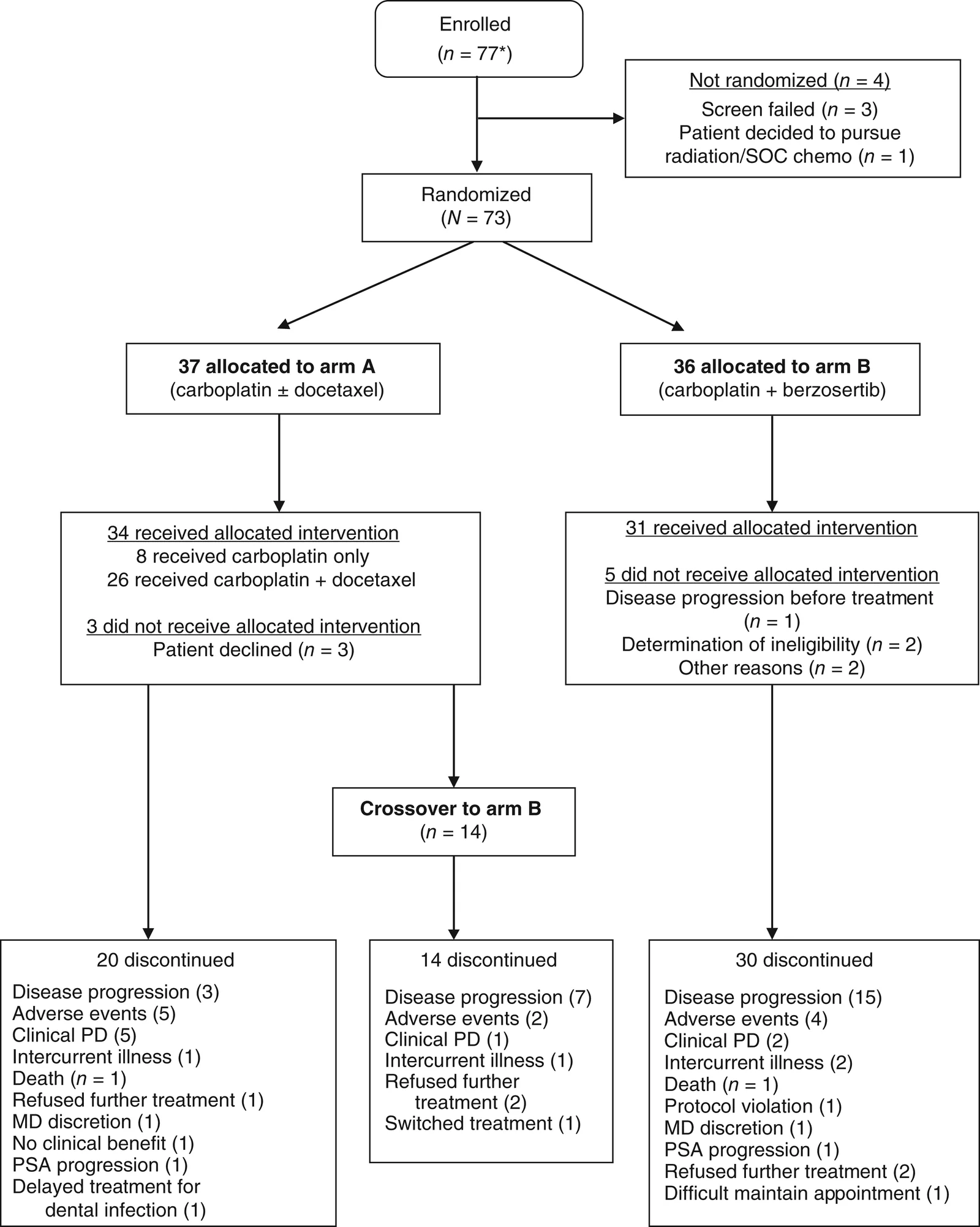
CONSORT diagram of enrolled, randomized, and treated patients in the study. MD, medical doctor; PD, progressive disease; SOC, standard of care. *Registration number was 78, one patient was registered twice.

At the time of the interim analysis, 73 patients had been randomized between June 2019 and July 2020, and 65 patients received protocol treatment (Fig. 1). Based on the interim analysis of these 65 patients, enrollment was halted due to futility.

Baseline characteristics for the 65 treated patients are shown in Table 1 with representativeness indicated in Supplementary Table S1. Thirty-four patients were treated on arm A (26 with docetaxel + carboplatin and 8 with carboplatin alone), and 31 were treated on arm B (berzosertib + carboplatin). The median number of prior systemic therapies [excluding androgen deprivation therapy (ADT), 5α-reductase inhibitors, and first-generation antiandrogens] was 4 (range, 2–8). The median number of cycles received in arm A was 3 (range, 1–20), and in arm B, it was 3 (range, 1–18). Fourteen patients initially randomized to arm A discontinued due to progression (7 for PSA progression, 2 for radiographic progression, and 5 for both radiographic and PSA progression) and crossed over to arm B. The median number of cycles received after cross-over was 3 (range, 1–9).

**Table 1.** Patient and disease characteristics at baseline.

Characteristic	Arm A: Carboplatin ± docetaxel (*N* = 34)		Arm B: Carboplatin + berzosertib (*N* = 31)	
	*N*	%	*N*	%
Race				
White	25	74	26	84
African American	3	9	3	10
Not reported	6	18	2	6
Measurable disease (from baseline lesion table)	20	59	17	55
ECOG PS				
0	12	35	10	32
1	21	62	17	55
2	1	3	4	13
Prior treatment				
Docetaxel	34	100	31	100
Cabazitaxel	14	41	13	42
AR pathway inhibitors^a^	34	100	31	100
Prior PARPi	3	9	3	10
	Median	IQR	Median	Range
Age at study entry	69	64–75	69	60–72
Baseline PSA, ng/mL	90	34–221	75	33–542
No. of prior systemic therapies^b^	4	3–4	3	3–5

Abbreviation: AR, androgen receptor.

a Abiraterone/apalutamide/darolutamide/enzalutamide.

b Excluding androgen deprivation therapy (ADT), 5α-reductase inhibitors, first-generation antiandrogens.

### Safety

All 65 treated patients reported TrAEs of any grade (Table 2; Supplementary Table S2). Grade 3 to 4 TrAEs were seen in 13 out of 34 (38%) of patients in arm A and 20 out of 31 (65%) in arm B. One patient in arm B experienced a grade 5 event of sepsis attributed to study treatment. Patients in arm B experienced a greater frequency of grade 3 to 4 thrombocytopenia (8/31, 26%) versus arm A (3/34, 9%). After cross-over from arm A to arm B, grade 3 TrAEs were seen in 4 out of 14 (29%) patients (Supplementary Table S3).

**Table 2.** TrAE observed in >10% of the 65 patients who received assigned protocol treatment (worst grade by patient and toxicity type).

​CTCAE term	Arm A^a^ Carboplatin ± docetaxel (*N* = 34)					Arm B Carboplatin + berzosertib (*N* = 31)				
	1	2	3	4	Total	1	2	3	4	Total
Platelet count decreased	4	9	3	​	16	6	7	5	3	21
Anemia	1	7	5	​	13	1	5	16	​	22
Fatigue	7	4	3	​	14	5	6	3	​	14
Nausea	8	2	​	​	10	7	5	​	​	12
Anorexia	5	3	​	​	8	7	3	​	​	10
White blood cell decreased	​	4	1	​	5	4	4	3	​	11
Neutrophil count decreased	3	1	3	1	8	4	1	2	​	7
Constipation	5	​	​	​	5	6	1	​	​	7
Aspartate aminotransferase increased	5	1	​	​	6	5	​	​	​	5
Diarrhea	1	4	1	​	6	4	​	​	​	4
Lymphocyte count decreased	2	​	1	​	3	1	3	2	​	6
Dysgeusia	4	2	​	​	6	3	​	​	​	3
Hypomagnesemia	1	1	​	​	2	4	​	1	1	6
Generalized muscle weakness	2	​	1	​	3	3	2	​	​	5
Dyspnea	1	​	​	​	1	4	2	​	​	6
Vomiting	3	1	​	​	4	3	​	​	​	3
Hyponatremia	2	1	​	​	3	4	​	​	​	4

a Arm A toxicities only included toxicities that occurred during arm A treatment (i.e., before patients’ cross-over to arm B).

### Efficacy

**Figure 2.**
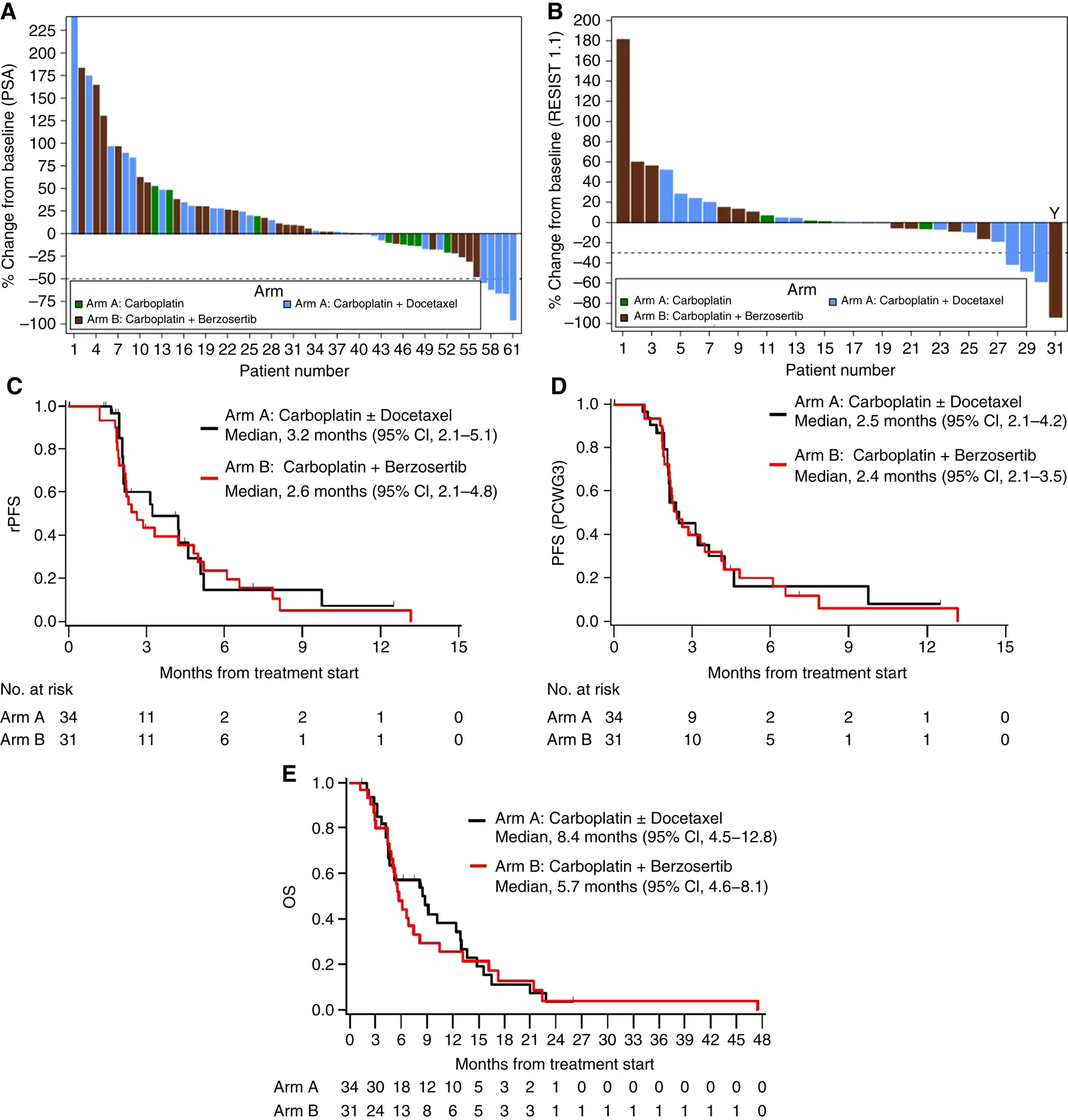
Clinical outcomes of berzosertib + carboplatin and docetaxel + carboplatin. **A** and **B,** Waterfall plots of (**A**) maximal PSA decline and (**B**) decline in measurable lesions by RECIST 1.1 from baseline. One patient in arm B (Y, far right) had an unconfirmed PR at cycle 8, followed by progressive disease. **C–E,** Kaplan–Meier estimates of (**C**) rPFS, (**D**) PFS by PCWG3 criteria (“no longer clinically benefiting,” i.e., radiographic or clinical/symptomatic progression), and (**E**) OS.

The primary endpoint, ORR, was 15% in arm A (5/34) and 0% in arm B (0/31; Fig. 2A and B). All responses in arm A were in patients who received docetaxel + carboplatin (5/26; 19%) with 0 out of 8 responses with carboplatin monotherapy. Of the five responders, two had both PSA and radiographic response, and three had PSA response only. Of note, two patients who achieved unconfirmed partial response (PR) were not counted as responders: One patient in arm B experienced unconfirmed PR at cycle 8 followed by disease progression (new soft tissue in bone seen on CT), and one patient in arm A (docetaxel + carboplatin) experienced unconfirmed PR at cycle 6, then discontinued treatment due to adverse events. Of the 14 patients who crossed over from arm A to arm B, 0 out of 14 experienced a response.

Other clinical endpoints were similar to numerically slightly inferior for arm B compared with arm A. Median rPFS was 3.2 (95% CI, 2.1–5.1) months in arm A and 2.6 (2.1–4.8) months in arm B [HR, 1.14 (0.62–2.1); Fig. 2C], whereas median PFS by PCWG3 criteria was 2.5 (2.1–4.2) months in arm A and 2.4 (2.1–3.5) months in arm B [HR, 1.05 (0.58–1.90); Fig. 2D]. Time to PSA progression was 2.1 (1.4–3.6) months in arm A and 1.4 (1–2.8) months in arm B [HR, 1.65 (0.88–3.09)]. In arm A, median OS was 8.4 (4.5–12.8) months with a 6-month OS of 57% (39%–72%), whereas in arm B, median OS was 5.7 (4.6–8.1) months with a 6-month OS of 44% (26%–61%; Fig. 2E). Fifty-five deaths were reported, and the median follow-up was 5.7 months (range, 1.3–26) among the 10 surviving patients.

### HRD assessment from tissue

**Figure 3.**
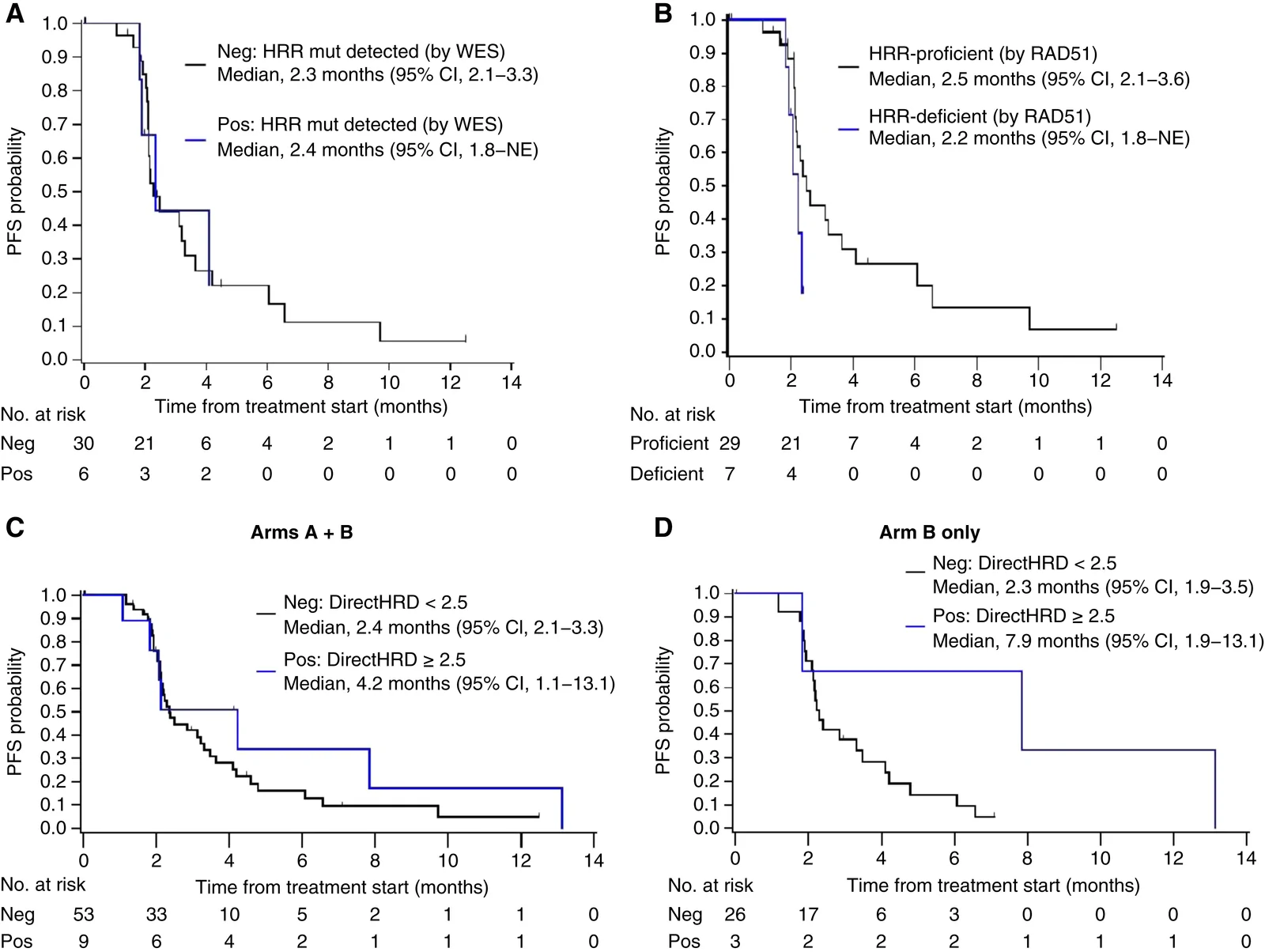
HRD status does not demonstrate a statistically significant correlation with clinical benefit in either arm. Kaplan–Meier plots for PFS per PCWG3 criteria with patients across both arms and stratified by (**A**) HRD status determined by deleterious DDR gene mutations identified by WES of tumor biopsy, (**B**) RAD51 foci–based IHC, (**C**) DirectHRD analysis of WGS of cfDNA using a cutoff of 2.5 in the overall cohort, and (**D**) DirectHRD analysis of WGS of cfDNA using a cutoff of 2.5 in arm B only. NE, not estimable.

A key secondary endpoint of the study was the relationship of “HRD” detected from the pretreatment tumor biopsy (as defined in the “Whole-exome sequencing” section of the “Patients and Methods”) with clinical endpoints. WES was successfully performed in 36 of 65 treated patients (55%). Deleterious variants in these DDR genes were detected in six patients: two with *CDK12*, two with *PALB2*, and one each with *BRCA1* (in conjunction with a *PALB2* variant), *ATM*, and *RAD54L* (Supplementary Fig. S1; Supplementary Table S4). There was no statistical difference in PFS for patients with or without these DDR gene mutations (Fig. 3A) despite all patients in the study being treated with carboplatin chemotherapy, which might be expected to be more active in the presence of DDR deficiency.

An orthogonal assay for HRR deficiency from tissue is RAD51 focus formation (30). We previously described a RAD51-based immunohistochemistry (IHC) assay that identifies HRR functional status (30) and correlates with olaparib sensitivity (36). Forty-one cases were evaluated for RAD51 focus formation, of which 7 were categorized as positive, 29 were categorized as negative, and 5 were categorized as indeterminate. Of the seven HRR-deficient cases by RAD51 focus formation, only two showed pathogenic variants from WES (*RAD54L* p.D183E and *PALB2* p.M296*), whereas the other DDR gene mutations did not confer loss of RAD51 focus formation (Supplementary Table S4). PFS for the cases considered functionally HRR-deficient by RAD51 focus formation was short despite treatment with carboplatin in all of these patients (Fig. 3B).

### HRD assessment from plasma

As tissue-based biomarkers were only able to be assessed in a subset of patients, we also collected plasma for circulating cfDNA profiling. We successfully isolated cfDNA from plasma for 64 of the 65 treated patients, encompassing 95 specimens in total. A comut plot depicting the variant summary is shown in Supplementary Fig. S1, and deleterious variants are listed in Supplementary Table S4. In general, the DDR mutations detected by WES from tissue were also detected in cfDNA, except for the *RAD54L* variant (which is not included in the sequencing panel) and the *PALB2* p.M296* variant. We conjectured that mutations detected in cfDNA that were not detected by WES from the matched tumor were likely subclonal, as the variant allele frequency as a proportion of TFx was <20% in these cases (except for one *ATM* variant seen in a sample in which TFx was very low).

Deeper WGS from cfDNA allowed for assessment of functional HRD using DirectHRD (35). This is an ultrasensitive scar-based classifier that makes use of a specific type of genomic scar—a small, microhomology deletion (mhDel) believed to be direct evidence of microhomology-mediated end joining in the absence of HR. This assay was successfully performed on 62 pretreatment specimens with matched germline available and TFx > 0. There was no difference in PFS for patients with and without detected HRD by this assay using the published cutoff of 1 for HRD score in the overall cohort (Supplementary Fig. S2A) or in arm B only (Supplementary Fig. S2B). A cutoff of 2.5 for DirectHRD score showed greater concordance with other orthogonal assays compared with a cutoff of 1 (Supplementary Table S4). Utilizing this more stringent cutoff demonstrates a nonsignificant (*P* = 0.365) suggestion of greater benefit from trial therapy with a high DirectHRD score in the overall cohort (Fig. 3C) and in arm B (Fig. 3D). Of the five HRR-deficient cases by RAD51 focus formation without detected DDR gene mutation, two were found to be HRD-positive by DirectHRD, providing further evidence that these represent *bona fide* HRD cases.

### ATM and replication stress biomarkers

**Figure 4.**
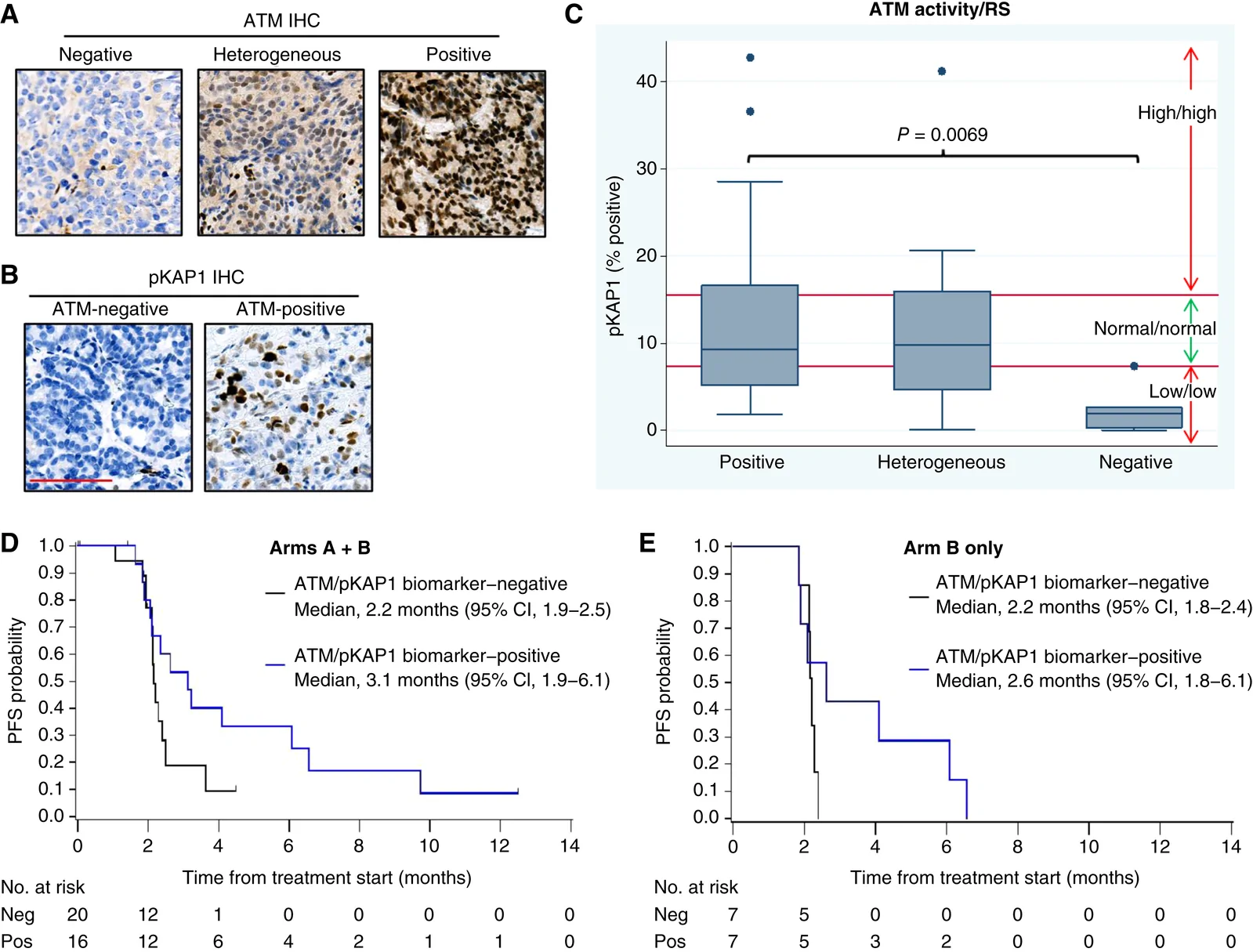
A combined ATM-RS biomarker identifies the best responders. **A** and **B,** Representative images of ATM (**A**) and pKAP1 (**B**) staining by IHC in tumor biopsy samples. The scale bar corresponds to 100 μm. **C,** Box plot showing pKAP1 levels in samples stratified by ATM status. Horizontal red lines depict the cutoff used to classify samples as ATM-RS–positive or ATM-RS–negative. **D** and** E,** Kaplan–Meier plots for PFS in patients across both arms (**D**) and in Arm B only (**E**), stratified by the combined ATM-RS classification.

We also performed IHC for ATM expression as a potential biomarker for benefit from berzosertib, as cells deficient in ATM expression have previously been shown to be sensitive to ATR inhibition (37–40). Representative images of ATM staining and interpretation are shown in Fig. 4A. A total of 40 specimens were evaluable for ATM expression, of which 22 were positive (14 in arm A and 8 in arm B), and 18 were negative or had heterogeneous expression (11 in arm A and 7 in arm B). Across both arms, patients with negative or heterogeneous ATM expression tended to have longer PFS than those with positive ATM expression (log-rank *P* = 0.117) although this difference did not reach statistical significance (Supplementary Fig. S3A). A similar trend was observed when the analysis was restricted to arm B alone (Supplementary Fig. S3B).

Serine 824 (S824) of KAP1 is a substrate of ATM, and phosphorylation levels of S824-KAP1 could serve as a biomarker of ATM activity (41–43). Additionally, ATR and DNA-PK can phosphorylate the serine 824 site of KAP1 under conditions of replication stress or genotoxic stress (44, 45). Thirty-two specimens were evaluable for pKAP1 IHC, with representative images and interpretations shown in Fig. 4B. Nine showed high pKAP1 levels (6 in arm A and 3 in arm B), and 23 demonstrated low levels (15 in arm A and 8 in arm B). There was no association between baseline levels of pKAP1 and PFS in this study (Supplementary Fig. S3C).

Because patients with negative or heterogeneous ATM expression derived greater benefit from therapy, we explored whether combining ATM and pKAP1 would have greater predictive power than either biomarker alone for identifying the best responders to a platinum-based regimen. Based on ATM and pKAP1 staining, the cohort was divided into three groups: (i) low ATM activity, (ii) normal ATM activity, and (iii) high replication stress (Fig. 4C). The low ATM activity group comprised tumors that were either ATM-negative or those with heterogeneous ATM expression and a percentage of pKAP1 positivity lower than that observed in the group of ATM-negative tumors. Tumors with high replication stress were defined as those with pKAP1 positivity greater than the 75th percentile irrespective of ATM status. These two groups were combined to form the ATM/pKAP1 biomarker–positive group. All other tumors were classified as ATM/pKAP1 biomarker–negative (normal) for both ATM activity and replication stress levels.

Interestingly, in the overall cohort, patients with tumors positive for this combined biomarker (i.e., either functionally deficient ATM or with high replication stress) experienced significantly longer PFS with treatment compared with those who were negative (i.e., functionally ATM-proficient and with low replication stress; log-rank *P* = 0.045; Fig. 4D). Similar results were observed in patients treated with arm B alone (Fig. 4E). These findings suggest that the ATM-pKAP1 combined biomarker is prognostic for improved outcomes with the carboplatin-based regimens utilized in this study.

## Discussion

In this phase II multicenter ETCTN study, we found that treatment with berzosertib + carboplatin led to less frequent clinical responses and similar PFS as a control regimen of carboplatin + docetaxel in a heavily pretreated, biomarker-unselected mCRPC population with metastatic disease amenable to biopsy. Serious adverse events were also more common in patients treated with berzosertib + carboplatin. To our knowledge, this is the first randomized trial of an ATR inhibitor in prostate cancer and the only prostate cancer–specific study combining this class of drug with carboplatin, which is already a commonly used agent in aggressive variant prostate cancers. Our finding that the combination of berzosertib with carboplatin did not demonstrate synergistic activity in patients compared with the combination with a taxane was unexpected based on preclinical studies. Previous trials of the ATR inhibitors ceralasertib (46), elimusertib (47), and camonsertib (48) as monotherapy have demonstrated modest clinical activity even in biomarker-selected patients with mCRPC. Notably, both ATM and DNA-PK activity may be activated in response to ATR inhibition, compensatory events that may limit ATR inhibitor efficacy (49, 50). In this regard, analysis of pATM and pDNA-PK by IHC will be of interest in future trials using ATR inhibitors, and appropriate biomarker selection and novel therapeutic combinations, such as with inhibitors of ATM and DNA-PK (51, 52), are likely to be required to improve clinical outcomes with this class of agent.

Findings from our trial provide important insights about drugs that synergize with carboplatin in mCRPC, which can guide clinical management and the design of future clinical trials in these patients. Specifically, our finding that the only responses seen in this study were in patients who received carboplatin and docetaxel was surprising because (i) all patients who received carboplatin plus docetaxel in this trial had previously progressed on docetaxel alone and (ii) the carboplatin dose of AUC4 given with docetaxel was lower than that used for carboplatin with berzosertib or carboplatin alone (AUC5). Because there are no prior randomized trials of carboplatin plus docetaxel compared with carboplatin alone, this trial provides compelling evidence that the combination of carboplatin with docetaxel is favored clinically over carboplatin monotherapy in a biomarker-unselected population, a finding that has important implications for clinical practice.

Our understanding of the mechanism by which carboplatin leads to prostate cancer cell death is incomplete: the primary hypothesis of our study that carboplatin would lead to DNA replication stress (through the generation of intra- and interstrand cross-links between nucleotide bases) that would then render prostate cancer cells ATR-dependent and vulnerable to ATR inhibition, even in cancers without DDR defects, was unsupported. One explanation could be that the ATR inhibitor used in this study, berzosertib, was ineffective at the dose tested. Due to dose-limiting overlapping toxicities of ATR inhibitors with platinum agents (53, 54), the recommended phase II dose of berzosertib (90 mg/m^2^ on days 2 and 9) in combination with carboplatin (28) is significantly lower than the dose of 210 mg/m^2^ on days 2 and 9 used in combination with gemcitabine (55) and with cisplatin (low dose) + veliparib (56), in which clinical responses were seen. As on-treatment biopsies were not performed in our study, we were unable to conduct pharmacodynamic assessments of berzosertib activity, such as the reduction of pCHK1 or the compensatory increase in phosphorylation of ATM substrates KAP1 and RAD50, to confirm adequate ATR inhibition. In a prior study of patients treated with berzosertib in combination with carboplatin AUC5, less reduction of pCHK1 was seen in four patients treated with berzosertib at 90 mg/m^2^ (−73%, −73%, −29%, +75%) compared with one patient treated with berzosertib at 120 mg/m^2^ (−94%; ref. 28).

Another explanation is that prior evidence of clinical benefit from carboplatin-containing regimens was due to synergy with docetaxel through mechanisms unrelated to DDR defects or the induction of replication stress. Indeed, a recent study (57) suggested that docetaxel sensitizes prostate cancer cells to carboplatin by affecting inflammatory pathways that make cells more likely to undergo apoptosis in response to carboplatin. These findings are relevant in designing trials of carboplatin-containing regimens in the future.

Our biomarker studies revealed that few patients with definitive HRD were enrolled. The lack of concordance between the assays and uncertainty around the biology they represent in mCRPC suggest that no individual assay is likely to encompass all patients who might benefit from DDR-targeted therapy. Even if a tissue-based test like the RAD51 focus formation assay represents a “gold standard,” this would not be universally feasible, as only 36 of the 65 treated patients had interpretable results despite the requirement for a pretreatment biopsy. Discrepancies between assays can be technical (due to detection limits or technical challenges) or biological due to (i) tumor heterogeneity (biopsy of a single site vs. cfDNA representing total tumor burden), (ii) assays reflecting different biology (e.g., HRR gene mutations and mhDels reflect the history of tumor HRD, whereas RAD51 focus formation reflects real-time functional status), (iii) the potential for disparate phenotypes conferred by different mutations in the same gene (e.g., due to the affected domain or whether a deleterious mutation results in complete loss of function vs. hypomorphic activity), and (iv) unassayed contributors to HRD (e.g., promoter methylation or gene expression changes).

Interestingly, the detection of “HRR” gene mutations showed limited predictive capacity for clinical benefit from carboplatin-based therapy. This result is consistent with findings from a recent study that was prematurely stopped due to limited activity of carboplatin monotherapy in patients with heavily pretreated, HRR-deficient mCRPC (58), as well as other trials that have demonstrated minimal activity of carboplatin monotherapy in non–BRCA HRR-mutated patients (59, 60). Although another prospective study of carboplatin in HRR-mutated mCRPC is pending (61), the mechanisms mediating response to this agent in non–BRCA-mutated mCRPC remain unclear and may not fully correlate with HRD.

In our study, most patients with tumor tissue amenable to profiling had tumors demonstrating either ATM deficiency (IHC-negative for ATM expression or functionally ATM-deficient with very low levels of pKAP1) or high replicative stress (indicated by very high pKAP1 levels). Trial participants with neither feature had a very poor response to carboplatin-based treatment. There is emerging evidence that genetic mutations in *ATM* may sensitize patients to other DNA-damaging systemic therapies for mCRPC, such as radium-223 (62) and ^177^Lu-PSMA (63), which is consistent with the suggestion that ATM deficiency confers vulnerability to carboplatin. Given the additional toxicity of carboplatin when added to a taxane, a biomarker to predict lack of benefit from this agent could have immediate clinical applicability.

Of most relevance in relation to existing treatments for prostate cancer is the identification of patients with likely HRD based on multiple orthogonal assays (RAD51 focus formation from tumor and DirectHRD from cfDNA) without detectable HRR gene mutations, as has been reported in other studies (64, 65). This suggests that some patients without detectable HRR gene mutations could benefit from a PARPi and, accordingly, that some of the benefit from PARP inhibition in the non–HRR-mutated populations in the PROfound trial of olaparib + abiraterone (66) and the TALAPRO-2 trial of talazoparib + enzalutamide (7) could be related to phenotypic HRD present in a subgroup of patients for which a clinical assay is an important clinical need.

### Conclusions

Berzosertib with carboplatin led to fewer clinical responses and more frequent serious adverse events compared with a control regimen of docetaxel with carboplatin in a randomized phase II study. No tissue or blood-based biomarker identified a subgroup with differential benefit from ATR inhibition. A combined biomarker, including ATM deficiency or high replicative stress, warrants exploration as a prognostic biomarker for patients planned for carboplatin treatment. Overall, our findings provide important preliminary data around biomarker selection for eligibility for trials of DDR-targeted therapies in the future.

## Supplementary Material

Supplementary Figure S1 Co-mut plot depicting variants in commonly altered genes in mCRPC (TP53, AR, PIK3CA, PTEN, RB1) and in HRR genes detected in tumor by whole exome sequencing (center triangles, blue) and in cfDNA by targeted panel sequencing at time point 1 (left triangles, purple) and time point 2 (right triangles, green).

Supplementary Figure S2 Kaplan-Meier curves for PFS with patients stratified by HRD status determined by DirectHRD analysis of WGS data from cfDNA.

Supplementary Figure S3 Kaplan-Meier curves for PFS stratified by ATM status by immunohistochemistry in tumor biopsy specimens, and scatter plot showing the relationship between PFS and % pKAP1 positivity in tumor biopsy specimens

Supplementary Table S1 Representativeness of Study Participants

Supplementary Table S2 TrAE among 65 patients who received assigned protocol treatment (worst grade by patient and toxicity type)

Supplementary Table S3 TrAE among 14 patients after crossover from Arm A to Arm B (worst grade by patient and toxicity type)

Supplementary Table S4 Summary of blood- and tissue-based biomarkers

## Data Availability

Individual data that underlie the results reported in this article, after deidentification (text, tables, figures, and appendices), can be made available upon reasonable request for investigators whose proposed use of the data has been approved. Requests should be directed to the National Cancer Institute (NCI) and will be subject to review and approval by the NCI and study leadership. Sequencing data are available under the Database of Genotypes and Phenotypes accession code phs003956.v1.p1.
